# Treatment of refractory Hailey-Hailey disease with oral upadacitinib and topical ruxolitinib 1.5% cream

**DOI:** 10.1016/j.jdcr.2025.05.052

**Published:** 2025-07-28

**Authors:** George Alhwayek, Maggie Sanders, Victoria Farley

**Affiliations:** a University of Nevada, Las Vegas School of Medicine, Las Vegas, Nevada; b Vivida Dermatology, Las Vegas, Nevada

**Keywords:** ATP2C1 mutation, dermatology case report, JAK inhibitors, refractory dermatologic conditions, ruxolitinib, upadacitinib

## Introduction

Hailey-Hailey disease (HHD), also known as benign familial chronic pemphigus, is a rare, inherited skin disorder characterized by recurrent blisters and erosions, particularly in intertriginous areas such as the groin, neck, lower back, and under the breasts.^1^ The disease is caused by mutations in the ATP2C1 gene, which encodes for a calcium pump in the Golgi apparatus, leading to impaired keratinocyte adhesion and resulting in the characteristic acantholysis seen in HHD.^2^ The autosomal dominant inheritance pattern often results in a family history of the condition, although with variable expressivity.^3^

HHD typically manifests in adulthood, although symptoms can appear earlier.^4^ The disease course is chronic with periods of exacerbation and remission, often triggered by sweating, minor trauma, and secondary infections.^5^ The condition significantly impacts the quality of life due to its chronic nature and the discomfort associated with flare-ups. HHD can be misdiagnosed as candidiasis, inverse psoriasis, tinea, impetigo, or contact dermatitis, which could result in improper treatment.^6^ Standard treatment options for HHD primarily focus on symptomatic relief, including the use of topical steroids, antibiotics, and antifungals to manage secondary infections. However, these treatments often provide only temporary relief and do not address the underlying pathophysiological mechanisms driving the disease.^7^

Recent advances in understanding the molecular pathways involved in HHD have led to the exploration of targeted therapies, particularly immunomodulators. Upadacitinib and ruxolitinib have emerged as potential therapeutic options due to their ability to inhibit the Janus kinase (JAK) pathway, a known pathway in the pathophysiology of HHD.^8^ Upadacitinib, an oral JAK1 inhibitor, is Food and Drug Administration–approved in treating various inflammatory conditions including rheumatoid arthritis, ulcerative colitis, and atopic dermatitis.^9^ In addition, ruxolitinib, a topical JAK1/2 inhibitor, is Food and Drug Administration–approved for treatment of atopic dermatitis.^10^ This case report presents the successful management of refractory HHD using a combination of oral upadacitinib and topical ruxolitinib cream, highlighting the potential of JAK inhibitors in treating this condition.

## Case report

**Fig 1:**
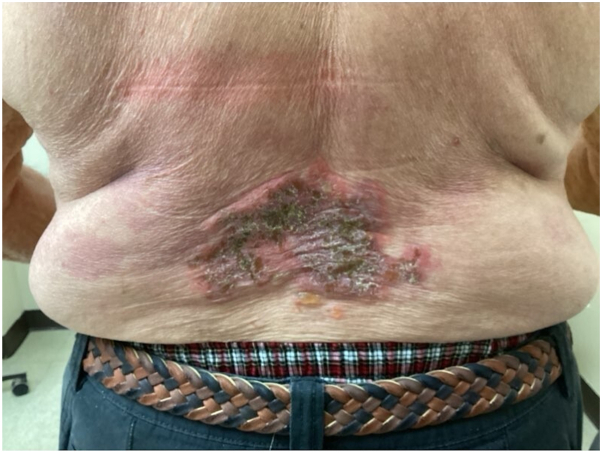
Image showing a flare-up of Hailey-Hailey disease on the patient's back after 5 months of remission.

**Fig 2:**
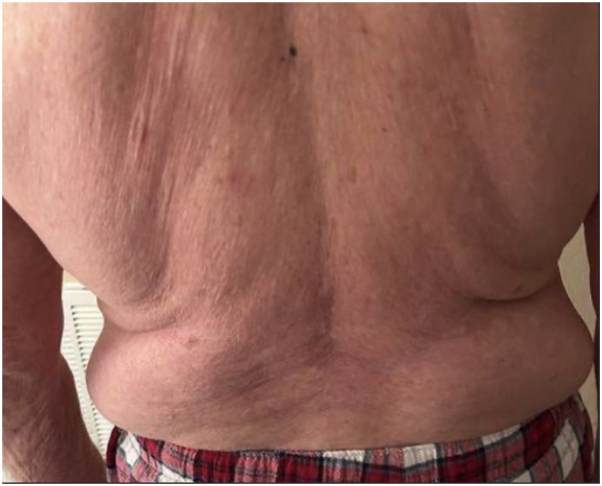
Image demonstrating significant improvement in redness and itchiness on the patient's back following the reintroduction of oral upadacitinib 15 mg daily.

The patient is an 84-year-old male with a significant past medical history of basal cell carcinoma, squamous cell carcinoma, and atherosclerotic cardiovascular disease began experiencing symptoms of HHD several years before the current case presentation. His condition was diagnosed by biopsy by a past provider and confirmed clinically with presentation of recurrent blisters and erosions in his groin and trunk. Prior to treatment with the JAK inhibitors, this patient experienced inadequate relief from steroids, doxycycline, sulfamethoxazole/trimethoprim, antifungals, intralesional kenalog, glycopyrrolate, and CO_2_ laser therapy. The patient was initially started on upadacitinib 15 mg daily, which resulted in significant improvement in symptoms within 1 month. After discontinuing Upadacitinib, he remained in remission without medication for 2 months until contracting COVID-19. Following the infection, he resumed Upadacitinib for 3 more months, experiencing another period of remission for 3 months until a flare-up occurred. Upadacitinib 15 mg daily was restarted with no improvement in symptoms; thus, dosage was increased to 30 mg daily. However, this led to side effects including dizziness and multiple falls. When upadacitinib 15 mg daily was reintroduced alongside ruxolitinib 1.5% cream, the patient achieved remission. After 5 months of maintaining remission with ruxolitinib cream alone, the patient experienced another flare-up on his back (Fig 1), as he had only been applying ruxolitinib cream to his groin. He then restarted on upadacitinib 15 mg daily, which led to significant improvement and cessation in symptoms (Fig 2).

This case report illustrates the successful use of upadacitinib in achieving periods of sustained remission in a male patient with refractory HHD. Th2-mediated interleukin-4 and interleukin-13 are associated with the JAK1 and signal transducer and activator of transcription 6 signaling pathway.^8^ The role of upadacitinib in JAK1 inhibition could possibly explain the efficacy observed in this case because it prevents inflammatory cytokines from using the JAK/signal transducer and activator of transcription pathway, a known part of the pathophysiology in HHD. Further research is warranted to explore the long-term safety and efficacy of JAK inhibitors in HHD, including studies that compare different dosing strategies and combinations of oral and topical therapies. Additionally, larger clinical trials are needed to establish standardized treatment protocols and to better understand the patient populations that would benefit the most from these interventions. While this case highlights the promise of targeted therapies in dermatology, it also emphasizes the importance of personalized treatment approaches. Each patient's unique medical history, treatment preferences, and response to medication should guide therapeutic decisions, particularly in managing rare and challenging conditions like HHD. In conclusion, the use of upadacitinib and ruxolitinib in this case has provided valuable insights into the potential for JAK inhibitors to effectively manage HHD. However, the balance between efficacy and safety remains crucial, and further studies are essential to optimize treatment outcomes for patients with this disease.

## Conflicts of interest

None disclosed.
